# Association of Sex With Postoperative Mortality Among Patients With Heart Failure Who Underwent Elective Noncardiac Operations

**DOI:** 10.1001/jamanetworkopen.2019.14420

**Published:** 2019-11-01

**Authors:** Aviva S. Mattingly, Benjamin J. Lerman, Rita Popat, Sherry M. Wren

**Affiliations:** 1 Stanford University School of Medicine, Stanford, California; 2 Department of Health Research and Policy, Stanford University School of Medicine, Stanford, California; 3 Surgical Service, Palo Alto Veterans Affairs Health Care System, Palo Alto, California; 4 Department of Surgery, Stanford University School of Medicine, Stanford, California

## Abstract

**Importance:**

Sex differences in postoperative outcomes in patients with heart failure (HF) have not been well characterized. Women generally experience a lower postoperative mortality risk after noncardiac operations. It is unclear if this pattern holds among patients with HF.

**Objectives:**

To determine if the risk of postoperative mortality is associated with sex among patients with HF who underwent noncardiac operations and to determine if sex is associated with the relationship between HF and postoperative mortality.

**Design, Setting, and Participants:**

This multisite cohort study used data from the US Department of Veterans Affairs Surgical Quality Improvement Project database for all patients who underwent elective noncardiac operations from October 1, 2009, to September 30, 2016, with a minimum of 1 year follow-up. The data analysis was conducted from May 1, 2018, to August 31, 2018.

**Exposures:**

Heart failure, left ventricular ejection fraction, and sex.

**Main Outcomes and Measures:**

Postoperative mortality at 90 days.

**Results:**

Among 609 735 patients who underwent elective noncardiac operations from 2009 to 2016, 47 997 patients had HF (7.9%; mean [SD] age, 68.6 [10.1] years; 1391 [2.9%] women) and 561 738 patients did not have HF (92.1%; mean [SD] age, 59.4 [13.4] years; 50 862 [9.1%] women). Among patients with HF, female sex was not independently associated with 90-day postoperative mortality (adjusted odds ratio [aOR], 0.97; 95% CI, 0.71-1.32). Although HF was associated with increased odds of postoperative mortality in both sexes compared with their peers without HF, the odds of postoperative mortality were higher among women with HF (aOR, 2.44; 95% CI, 1.73-3.45) than men with HF (aOR, 1.64; 95% CI, 1.54-1.74), suggesting that HF may negate the general protective association of female sex with postoperative mortality (*P* for interaction of HF × sex = .03). This pattern was consistent across all levels of left ventricular ejection fraction.

**Conclusions and Relevance:**

Although HF was associated with increased odds of postoperative mortality in both sexes compared with their peers without HF, the odds of postoperative mortality were higher among women with HF than men with HF, suggesting that HF may negate the general protective association of female sex with postoperative mortality risk in noncardiac operations.

## Key Points

**Question:**

Is sex associated with the risk of postoperative mortality among patients with heart failure?

**Findings:**

In this cohort study including 609 735 patients who underwent elective noncardiac operations, sex was not independently associated with postoperative mortality among patients with heart failure. Although HF was associated with increased odds of postoperative mortality in both sexes compared with their peers without HF, the odds of postoperative mortality were higher among women with HF than men with HF.

**Meaning:**

Heart failure may be associated with negating the general protective association of female sex with postoperative mortality in noncardiac operations.

## Teaser

This cohort study evaluates whether sex is associated with postoperative mortality among patients with heart failure who underwent elective noncardiac operations.

## Introduction

Across the world and in the United States, women are known to have a higher life expectancy than men.^1^ The most recent estimates for the US population indicate that life expectancy for women is 82.3 years, compared with 77.8 years for men.^2^ Women have better survivorship across a variety of specific settings, including trauma, famine, and epidemics, suggesting that female sex plays a protective role.^3,4^ This sex difference in mortality has been shown to be especially prominent among hospitalized patients.^5^

The protective effect associated with female sex remains relevant among the population of patients undergoing surgical procedures. There are significant associations of female sex with reduced postoperative mortality risk in patients who undergo noncardiac operations.^6^ While surgical studies explore this relationship broadly, we are interested in whether heart failure (HF) is associated with this well-characterized postoperative female mortality advantage.

Heart failure is a known risk factor for postoperative mortality, and patients with HF have been shown to experience worse postoperative outcomes compared with patients without HF.^7,8,9,10^ Heart failure is a complex and heterogeneous syndrome, and different demographic groups have distinct patterns of clinical presentation.^11,12^ This is particularly well described for sex, in that women with HF tend to be diagnosed at a younger age, are more likely to have preserved systolic function, and receive fewer medications compared with men.^13,14,15,16,17,18^ Although the different presentations of women and men with HF are well described, to our knowledge, the association of these clinical differences with disparities in surgical outcomes is unknown. The population of people with HF in the United States is estimated to increase to more than 8 million people by 2030.^19^ As such, a fundamental understanding of HF and its potential to affect the paradigm of the female mortality advantage is critical to perioperative decision-making for women.

This study used the well-characterized Veterans Affairs Surgical Quality Improvement Project (VASQIP) database to perform a large-scale cohort study investigating the combined association of sex and HF with risk of postoperative mortality in patients who underwent noncardiac operations. The purpose of this study was to assess sex differences in postoperative outcomes within the HF population and determine whether the operative risk associated with HF in patients who underwent noncardiac operations differed between women and men. We hypothesized that patients with HF would experience similar trends as those observed in the general population: that women who underwent noncardiac operations would be associated with lower mortality rates than men at 90 days.

## Methods

This study was approved by the Stanford University institutional review board and the US Department of Veterans Affairs (VA), and a waiver of informed consent was obtained because data were deidentified. This study was reported following the Strengthening the Reporting of Observational Studies in Epidemiology (STROBE) reporting guidelines.

### Design

This study is a retrospective cohort study from October 1, 2009, to September 30, 2016, with a minimum of 1 year follow-up (final study follow-up, September 1, 2017) using the VASQIP database. The cohort included all patients who underwent elective, noncardiac operations at VA hospitals during the study. Analysis took place between May 1, 2018, and August 31, 2018.

### Data Sources

Two national VA databases were used to assemble the cohort, as previously described.^10^ Data were primarily abstracted from VASQIP. When certain pertinent information was not available from VASQIP, the VA Corporate Data Warehouse, which extracts data directly from the VA electronic medical records system, was used. Medical comorbidities for each participant were extracted from VASQIP (eg, hypertension, stroke, chronic obstructive pulmonary disease, peripheral vascular disease, or disseminated cancer) and from VA Corporate Data Warehouse diagnostic codes (eg, atrial fibrillation, diabetes mellitus, asthma, aortic stenosis, mitral regurgitation, or pulmonary hypertension). Left ventricular ejection fraction (LVEF) data were obtained from echocardiogram reports using a previously validated natural language processing algorithm.^20,21^

Inclusion criteria were all VASQIP-sampled operative procedures performed between fiscal years 2009 to 2016 with minimum follow-up of 1 year, and final date of follow-up by September 1, 2017. Only the first operative procedure for each patient was included for analysis. Cardiac operations, emergent operations, and other minor operative procedures (eg, cystoscopy, bronchoscopy, colonoscopy, endoscopy, and other noninvasive procedures) were excluded. All eligible operations were classified by the VA Surgical Complexity Matrix, which assigns a complexity level of standard, intermediate, or complex.^10,22,23^ Surgical complexity was included as a covariate in the final multivariable models.

### Classification of HF and LVEF

Patients were classified as having HF if they had at least 1 inpatient admission or at least 2 outpatient clinic visits with a diagnosis of HF (by *International Classification of Diseases, Ninth Revision*^24^ code) within 3 years of the index operation, as previously described.^10,25^ Patients were excluded if HF was not the ultimate diagnosis after an outpatient visit. Patients with HF were further stratified by LVEF into 2 groups based on the American College of Cardiology treatment guidelines^26^: at least 40% or less than 40%.

### Outcome

The primary outcome of this study was all-cause 90-day postoperative mortality. Date of death was determined via Social Security Administration Death Master Files.

### Statistical Analysis

Demographic and clinical characteristics of female and male patients with and without HF were compared with χ^2^ tests for categorical variables and unpaired *t* tests for continuous variables. Odds ratios (ORs) were generated using simple or multivariable logistic regression, and 95% CIs were computed with Wald χ^2^. Potential confounders were assessed based on a priori knowledge and the literature.^27,28^ The following factors were considered: sex, self-reported race/ethnicity, age, body mass index (BMI, calculated as weight in kilograms divided by height in meters squared), smoking, alcohol use, hypertension, atrial fibrillation, diabetes, coronary artery disease, history of stroke, asthma, chronic obstructive pulmonary disease, peripheral vascular disease, disseminated cancer, surgical complexity level, American Society of Anesthesiologists (ASA) class, VA facility, preoperative creatinine level, and preoperative hematocrit. Covariates that altered the exposure parameter estimate by 10% or more were included in the final model.

Race/ethnicity is associated with long-term survival in patients with HF and was thus included as a covariate.^29^ Ethnicity classification was determined by the patient and was based on a fixed-categories questionnaire.

The final analysis consisted of 2 mixed-effects logistic regression models with random intercepts to account for clustering by VA facility. The first model compared the postoperative mortality risk of women and men with HF. The second model compared the postoperative mortality rates of patients with and without HF stratified by sex. Multiplicative interaction was tested between the primary exposure (HF, yes or no) and sex (female or male). *P* for interaction was calculated using the likelihood ratio test.

The proportion of missing nonlaboratory, nonimaging covariates was less than 1%. Missing observations were excluded from the analysis. Missing preoperative laboratory values (6%-7%) were imputed with a single conditional imputation approach using age- and sex-adjusted norms. Left ventricular ejection fraction as measured by echocardiogram was missing in 2.9% of patients with HF, and patients with missing LVEF were excluded from subset analyses pertaining to left ventricular systolic function. SAS statistical software version 9.4 (SAS Institute) was used for all analyses. *P* values were 2-sided, and statistical significance was set at *P* less than .05.

## Results

All 805 432 procedure records in the VASQIP database were sampled and analyzed for eligibility, encompassing 795 778 unique patients. Of these, 614 308 patients met the procedure eligibility criteria. We excluded 4573 records (0.7%) for missing nonlaboratory data values, and the remaining 609 735 patients (mean [SD] age, 60.1 [10.1] years; 557 482 [91.4%] men) were included in the final analysis. Among them, 47 997 patients had HF (7.9%; mean [SD] age, 68.6 [10.1] years; 1391 [2.9%] women) and 561 738 patients did not have HF (92.1%; mean [SD] age, 59.4 [13.4] years; 50 862 [9.1%] women). The 5 most common procedures by sex are listed in the eTable in the Supplement. Of 52 253 women included, 1391 (2.7%) had a clinical history of HF (Table 1). Women with HF compared with men with HF were more likely to be younger (mean [SD] age, 61.7 [12.2] years vs 68.8 [9.9] years; *P* < .001) and have higher BMI (31.9 vs 29.8; *P* < .001) and less likely to regularly consume alcohol at the time of the index operation (1.3% vs 5.4%; *P* < .001). Rates of smoking did not differ by sex. These associations of sex with BMI, alcohol use, and smoking status were similar when comparing women and men without HF (Table 1).

**Table 1.** Demographic Characteristics and Medical History of Women and Men With and Without HF

Characteristic	Patients With HF			Patients Without HF		
	No. (%)		*P* Value	No. (%)		*P* Value
	Women (n = 1391)	Men (n = 46 606)		Women (n = 50 862)	Men (n = 510 876)	
Ethnicity						
Non-Hispanic white	830 (60.0)	41 591 (67.8)	<.001	27 851 (54.8)	334 711 (65.6)	<.001
Non-Hispanic black	354 (25.5)	7467 (16.02)		12 398 (24.4)	73 831 (14.5)	
Hispanic	34 (2.4)	1709 (3.7)		2248 (4.4)	23 964 (4.7)	
Other	11 (0.8)	379 (0.8)		1093 (2.2)	5816 (1.1)	
Not reported	162 (11.7)	5460 (11.7)		7272 (14.3)	72 554 (14.2)	
Age, mean (SD), y	61.7 (12.2)	68.8 (9.9)	<.001	48.4 (12.7)	60.5 (13.0)	<.001
BMI, mean (SD)	31.9 (7.9)	29.8 (6.9)	<.001	30.1 (6.6)	28.9 (5.7)	<.001
Current smoker	389 (28.0)	12 697 (27.2)	.55	14 972 (29.4)	167 742 (32.8)	<.001
Current alcohol use	18 (1.3)	2495 (5.4)	<.001	1126 (2.2)	40 467 (7.9)	<.001
ASA class						
1	6 (0.4)	21 (0.1)	<.001	3248 (6.4)	13 826 (2.7)	<.001
2	137 (9.9)	1312 (2.8)		25 511 (50.2)	151 272 (29.6)	
3	984 (70.7)	30 271 (65.0)		21 328 (41.9)	316 429 (62.0)	
4	264 (19.0)	14 889 (32.0)		771 (1.5)	29 194 (5.7)	
5	0 (0)	113 (0.2)		4 (0.0)	155 (0.0)	
Comorbidity						
Hypertension	1079 (77.6)	40 998 (88.0)	<.001	18 576 (35.5)	306 342 (60.0)	<.001
Atrial fibrillation	290 (20.9)	18 708 (40.1)	<.001	1146 (2.3)	47 999 (9.4)	<.001
Diabetes	704 (50.6)	27 628 (59.3)	<.001	10 234 (20.1)	165 406 (32.4)	<.001
Coronary artery disease	572 (41.1)	31 587 (67.8)	<.001	3039 (6.0)	107 212 (21.0)	<.001
History of stroke	135 (9.7)	6079 (13.0)	<.001	945 (1.9)	24 317 (4.8)	<.001
Asthma	359 (25.8)	4012 (8.6)	<.001	9073 (17.8)	34 637 (6.8)	<.001
Chronic obstructive pulmonary disease	318 (22.9)	13 262 (28.5)	<.001	2632 (5.2)	58.078 (11.4)	<.001
Peripheral vascular disease	66 (4.7)	5545 (11.9)	<.001	285 (0.6)	16 306 (3.2)	<.001
Disseminated cancer	16 (1.2)	673 (1.4)	.36	225 (0.4)	5205 (1.0)	<.001
Laboratory values						
Creatinine, median (IQR), mg/dL^a^	0.90 (0.40)	1.12 (0.60)	<.001	0.80 (0.2)	1.0 (0.3)	<.001
Hematocrit, mean (SD), %^b^	37.3 (5.0)	38.1 (6.1)	<.001	39.2 (3.7)	41.8 (4.8)	<.001
Surgical complexity						
Standard	738 (53.1)	24 589 (52.8)	.30	34 281 (67.4)	307 468 (60.2)	<.001
Intermediate	628 (45.5)	20 881 (44.8)		16 018 (31.5)	193 004 (37.8)	
Complex	25 (1.8)	1136 (2.4)		563 (1.1)	10 404 (2.0)	

Abbreviations: ARB, angiotensin receptor blocker; ASA, American Society of Anesthesiologists; BMI, body mass index (calculated as weight in kilograms divided by height in meters squared); HF, heart failure; IQR, interquartile range.

SI conversion factors: To convert creatinine to micromoles per liter, multiply by 88.4; hematocrit to proportion of 1.0, multiply by 0.01.

^a^ Missing data for 42 072 patients (6.9%).

^b^ Missing data for 44 511 patients (7.3%).

Most women and men with HF were classified as ASA class 3 (70.7% vs 65.0%). However, a higher percentage of women than men were classified as ASA class 2 (9.9% vs 2.8%; *P* < .001), and a lower percentage of women than men were classified as ASA class 4 (19.0% vs 32.0%; *P* < .001). Women with HF, compared with men with HF, had lower rates of hypertension (77.6% vs 88.0%; *P* < .001), atrial fibrillation (20.9% vs 40.1%; *P* < .001), diabetes (50.6% vs 59.3%; *P* < .001), coronary artery disease (41.1% vs 67.8%; *P* < .001), chronic obstructive pulmonary disease (22.9% vs 28.5%; *P* < .001), and peripheral vascular disease (4.7% vs 11.9%; *P* < .001). Women with HF had higher rates of asthma than men with HF (25.8% vs 8.6%; *P* < .001). Women and men with HF received a similar distribution of standard, intermediate, and complex procedures (Table 1).

Left ventricular ejection fraction data were available for 1331 women with HF (95.7%) and 45 256 men with HF (98.3%) (Table 2). Women with HF were more likely than men with HF to have preserved LVEF (ie, LVEF ≥50%) (79.2% vs 61.2%; *P* < .001) and less likely to have severely reduced LVEF (ie, LVEF <30%) (5.0% vs 9.1%; *P* < .001). Women with HF were also less likely than men with HF to be receiving β-blockers (81.9% vs 92.0%; *P* < .001) or angiotensin-converting enzyme inhibitors or angiotensin receptor blockers (82.5% vs 92.6%; *P* < .001) (Table 2).

**Table 2.** Clinical Characteristics of Patients With Heart Failure

Characteristic	No. (%)	
	Women	Men
LVEF^a^		
Preserved	1054 (79.2)	27 688 (61.2)
Mildly reduced	123 (9.4)	7489 (16.6)
Moderately reduced	88 (6.6)	5960 (13.2)
Severely reduced	66 (5.0)	4119 (9.1)
Medications		
β-blocker	1139 (81.9)	42 891 (92.0)
ACE inhibitor or ARB	1148 (82.5)	43 132 (92.6)
Potassium-sparing diuretic	514 (36.7)	16 074 (34.5)

Abbreviations: ACE, angiotensin-converting enzyme; ARB, angiotensin receptor blocker; LVEF, left ventricular ejection fraction.

^a^ Includes 46 587 patients, of whom 82% had echocardiogram readings within 1 year of index operation. Does not include 2.9% of patients with missing LVEF data. Preserved indicates 50% or higher; mildly reduced, 40 to 49%; moderately reduced, 30 to 39%; and severely reduced, less than 30%.

Women and men with HF experienced crude 90-day postoperative mortality risks of 3.59% and 5.55%, respectively. This difference was not statistically significant after multivariable adjustment for demographic, clinical, and surgical complexity factors (adjusted OR [aOR], 0.97; 95% CI, 0.71-1.32) (Table 3). Age, preoperative hematocrit, and ASA class were all significant confounders of this association.

**Table 3.** Association of Sex With 90-Day Postoperative Mortality in Patients With Heart Failure

Sex	No.	Mortality (%)	Odds Ratio (95% CI)	
			Crude	Adjusted^a^
Men	46 606	5.55	1 [Reference]	1 [Reference]
Women	1391	3.59	0.63 (0.48-0.85)	0.97 (0.71-1.32)

^a^ Multivariable mixed-effects logistic regression model adjusted for age, body mass index, alcohol consumption, comorbidities (hypertension, atrial fibrillation, diabetes, coronary artery disease, stroke, chronic obstructive pulmonary disease, and peripheral vascular disease), preoperative creatinine level, preoperative hematocrit, American Society of Anesthesiologists class, surgical complexity, and US Department of Veterans Affairs facility.

Although HF was significantly associated with postoperative mortality in both sex compared with their peers without HF, the odds of postoperative mortality were significantly higher in women with HF (aOR, 2.44; 95% CI, 1.73-3.45) than in men with HF (aOR, 1.64; 95% CI, 1.54-1.74), and the *P* value for multiplicative interaction (HF × sex) was statistically significant (*P* for interaction = .03) (Table 4). This pattern was consistent after further subdividing the HF population by LVEF. In both sexes, patients with HF and LVEF of 40% or higher and patients with HF and LVEF of less than 40% all experienced higher 90-day postoperative mortality risk compared with those without HF (women with LVEF ≥40%: aOR, 2.31; 95% CI, 1.58-3.77; women with LVEF <40%: aOR, 3.72; 95% CI, 1.68-7.34; men with LVEF ≥40%: aOR, 1.50; 95% CI, 1.40-1.60; men with LVEF <40%: aOR, 2.03; 95% CI, 1.85-2.23) (Table 5).

**Table 4.** Association of HF With 90-Day Postoperative Mortality by Sex

Sex	No.	Mortality (%)	Odds Ratio (95% CI)	
			Crude	Adjusted^a^
Women				
Without HF	50 862	0.35	1 [Reference]	1 [Reference]
With HF	1391	3.59	10.74 (7.81-14.77)	2.44 (1.73-3.45)
Men				
Without HF	510 876	1.33	1 [Reference]	1 [Reference]
With HF	46 606	5.55	4.42 (4.22-4.62)	1.64 (1.54-1.74)

Abbreviation: HF, heart failure.

^a^ Multivariable mixed-effects logistic regression model adjusted for sex, age, body mass index, alcohol consumption, comorbidities (hypertension, atrial fibrillation, diabetes, coronary artery disease, stroke, chronic obstructive pulmonary disease, and peripheral vascular disease), preoperative creatinine level, preoperative hematocrit, American Society of Anesthesiologists class, and surgical complexity, and US Department of Veterans Affairs facility.

**Table 5.** Association of Heart Failure With 90-Day Postoperative Mortality by Sex and LVEF

Sex	No.	Mortality, %	Odds Ratio (95% CI)	
			Crude	Adjusted^a^
Women				
Without HF	50 862	0.35	1 [Reference]	1 [Reference]
With HF				
LVEF ≥40%	1177	3.40	10.12 (7.14-14.34)	2.31 (1.58-3.77)
LVEF <40%	154	5.84	17.90 (9.00-35.63)	3.72 (1.68-7.34)
Men				
Without HF	510 876	1.33	1 [Reference]	1 [Reference]
With HF				
LVEF ≥40%	35 177	5.00	3.97 (3.73-4.16)	1.50 (1.40-1.60)
LVEF <40%	10.079	7.32	5.94 (5.49-6.43)	2.03 (1.85-2.23)

Abbreviations: HF, heart failure; LVEF, left ventricular ejection fraction.

^a^ Multivariable mixed-effects logistic regression model adjusted for sex, age, body mass index, alcohol consumption, comorbidities (hypertension, atrial fibrillation, diabetes, coronary artery disease, stroke, chronic obstructive pulmonary disease, and peripheral vascular disease), preoperative creatinine level, preoperative hematocrit, American Society of Anesthesiologists class, surgical complexity, and US Department of Veterans Affairs facility.

## Discussion

In this cohort study including more than 50 000 female veterans, patients with HF of both sexes experienced equivalent 90-day postoperative mortality risk, which disproved our hypothesis that women would be associated with lower mortality rates than men. Although HF was associated with increased odds of postoperative mortality in both sexes compared with their peers without HF, the odds of postoperative mortality were higher among women with HF than men with HF. Although female sex is generally associated with better postoperative mortality after noncardiac operations, this analysis suggests that this pattern did not hold in the subpopulation of women with HF. The presence of HF as an operative risk factor may act as a type of equalizer associated with negating the protective association of female sex with postoperative mortality observed among the general population.

Although the percentage of women in this study was relatively small due to the use of the VA data set, the absolute number of female patients with HF included in this study was among the largest cohorts investigating postoperative mortality in the context of noncardiac operations, to our knowledge. While previous studies have shown that women and men with HF have similar postoperative mortality,^11^ this study is one of the only studies, to our knowledge, to use a cohort of patients without HF for comparison. We were able to examine the disproportionate association of HF with mortality among women based on this comparison group, and we found that HF was associated with higher odds of postoperative mortality among women than among men.

The clinical profiles of women with HF who underwent noncardiac operations within the VA health care system were similar to those of previous epidemiological profiles of women in the general HF population. In particular, women were less likely than men to present with additional medical comorbidities or reduced LVEF. However, despite these differences, our findings remained consistent after further subdividing patients with HF by LVEF.

Consistent with a 2010 study^30^ that found lower LVEF was an independent risk factor for postoperative mortality, our data show that lower LVEF was associated with higher 90-day postoperative mortality in both sexes. These findings were consistent when patients with HF were stratified into LVEF of 40% or more vs LVEF of less than 40%. Furthermore, the increased risk associated with HF among women remained consistent regardless of LVEF group.

Differences in HF management may contribute to our observed sex-based disparity in the association of HF with postoperative mortality. In our study population, women with HF were less likely than men with HF to be prescribed standard HF medications (ie, angiotensin-converting enzyme inhibitors, angiotensin receptor blockers, and β-blockers). This difference could be attributed to the differences seen in comorbidities between women and men, given that we found lower rates of hypertension, diabetes, and coronary artery disease, as well as a higher prevalence of preserved LVEF, in women. Trends in medication practices are likely multifactorial. However, it is possible that these medications, particularly β-blockers, exert a survival benefit in the perioperative period. Thus, the higher odds of postoperative mortality associated with HF in women may be mitigated by optimizing medical management prior to operation. Further research is indicated on this matter.

### Limitations

This study has some limitations. There was a possible selection bias in that all patients underwent operations in this cohort and no data were available on patients who were evaluated for a surgical procedure but who did not then undergo one. Therefore, there are no data on patients with HF who did not undergo an operation to use as a comparison for baseline mortality rates.

Second, as with any observational study, there was a risk of unmeasured confounding factors that may have, if accounted for, negated the apparent contribution of HF as an independent risk factor associated with postoperative mortality. To address this, a 2019 study by Lerman et al^10^ that was conducted using this data set calculated E-values as a sensitivity analysis to determine the likelihood that an unmeasured confounder could exist that would negate the observed association of HF with postoperative mortality. The E-value represents the minimum independent association of an unmeasured confounder with the relevant exposure and outcome for that confounder to fully attenuate the observed exposure-outcome association to the null. They concluded that such an unmeasured confounder was unlikely to exist, as the range of point estimates for the ORs for all the known risk factors available in the data were substantially lower than the calculated E-value.^10^

Third, the generalizability of this study is limited because the VA population of women may not have been representative of the general population in the United States. For example, this cohort had more than 2-fold the rates of smoking; in the general population, 12% of women smoke,^31^ compared with 28% to 29% of women in this study. Similarly, the prevalence of hypertension among women in the United States is 28% and the prevalence of diagnosed diabetes is 9%, while the VA non-HF cohort of women had increased prevalence of hypertension at 35.5% and more than 2-fold the prevalence of diabetes at 20.1%.^32,33^

Fourth, while this study included one of the largest cohorts of female patients with HF undergoing noncardiac operations in the literature, to our knowledge, the difference in sample size between women and men may have affected the study findings. It is possible that if the VA health care database had a larger sample of women, there may have been changes in the confidence intervals found during this analysis.

## Conclusions

This cohort study found that although HF was associated with increased odds of postoperative mortality in both sexes compared with their peers without HF, the odds of postoperative mortality were higher among women with HF than men with HF, even after controlling for LVEF. This suggests that HF may negate the general protective association of female sex with postoperative mortality in noncardiac operations.

## Notes

Supplement. **eTable.** Most Frequent Procedures Performed in Women and Men
