# Supplementary material for: Association of Sex With Postoperative Mortality Among Patients With Heart Failure Who Underwent Elective Noncardiac Operations
Source: JAMA Netw Open. 2019 Nov 1;2(11):e1914420. doi: 10.1001/jamanetworkopen.2019.14420 (PMC6826642; doi:10.1001/jamanetworkopen.2019.14420)
Supplement: Supplement. — eTable. Most Frequent Procedures Performed in Women and Men [file jamanetwopen-2-e1914420-s001.pdf]

Supplementary Online Content

Mattingly AS, Lerman BJ, Popat R, Wren SM. Association of sex with postoperative mortality among patients with heart failure who underwent elective noncardiac operations. *JAMA Netw Open*. 2019;2(11):e1914420. doi:10.1001/jamanetworkopen.2019.14420

eTable. Most Frequent Procedures Performed in Women and Men

This supplementary material has been provided by the authors to give readers additional information about their work.

© 2019 Mattingly AS et al. *JAMA Network Open.*

eTable. Most Frequent Procedures Performed in Women and Men

Procedure	No. (%)
Women	
Laparoscopic Cholecystectomy	4,501 (8.7)
Arthroplasty, Total Knee	2,057 (4.0)
Total Abdominal Hysterectomy	2,035 (3.9)
Arthroscopic Meniscectomy	1,712 (3.3)
Reduction Mammoplasty	1,515 (2.9)
Men	
Inguinal Hernia Repair	49,701 (9.0)
Total Knee Arthroplasty	30,311 (5.5)
Laparoscopic Cholecystectomy	18,588 (3.4)
Arthroscopic Meniscectomy	17,822 (3.2)
Total Hip Arthroplasty	16,287 (3.0)

© 2019 Mattingly AS et al. *JAMA Network Open.*
